# Life Cycle, Ultrastructure, and Phylogeny of New Diplonemids and Their Endosymbiotic Bacteria

**DOI:** 10.1128/mBio.02447-17

**Published:** 2018-03-06

**Authors:** Daria Tashyreva, Galina Prokopchuk, Jan Votýpka, Akinori Yabuki, Aleš Horák, Julius Lukeš

**Affiliations:** a Biology Centre, Institute of Parasitology, Czech Academy of Sciences, České Budějovice (Budweis), Czech Republic; b Faculty of Sciences, Charles University, Prague, Czech Republic; c Department of Marine Diversity, Japan Agency for Marine-Earth Science and Technology, Yokosuka, Japan; d Faculty of Science, University of South Bohemia, České Budějovice (Budweis), Czech Republic; Duke University

**Keywords:** *Holosporales*, diplonemid, endosymbionts, life cycle, ultrastructure

## Abstract

Diplonemids represent a hyperdiverse and abundant yet poorly studied group of marine protists. Here we describe two new members of the genus *Diplonema* (Diplonemea, Euglenozoa), *Diplonema japonicum* sp. nov. and *Diplonema aggregatum* sp. nov., based on life cycle, morphology, and 18S rRNA gene sequences. Along with euglenozoan apomorphies, they contain several unique features. Their life cycle is complex, consisting of a trophic stage that is, following the depletion of nutrients, transformed into a sessile stage and subsequently into a swimming stage. The latter two stages are characterized by the presence of tubular extrusomes and the emergence of a paraflagellar rod, the supportive structure of the flagellum, which is prominently lacking in the trophic stage. These two stages also differ dramatically in motility and flagellar size. Both diplonemid species host endosymbiotic bacteria that are closely related to each other and constitute a novel branch within *Holosporales*, for which a new genus, “*Candidatus* Cytomitobacter” gen. nov., has been established. Remarkably, the number of endosymbionts in the cytoplasm varies significantly, as does their localization within the cell, where they seem to penetrate the mitochondrion, a rare occurrence.

## IMPORTANCE

We describe the morphology, behavior, and life cycle of two new *Diplonema* species that established a relationship with two *Holospora*-like bacteria in the first report of an endosymbiosis in diplonemids. Both endosymbionts reside in the cytoplasm and the mitochondrion, which establishes an extremely rare case. Within their life cycle, the diplonemids undergo transformation from a trophic to a sessile and eventually a highly motile swimming stage. These stages differ in several features, such as the presence or absence of tubular extrusomes and a paraflagellar rod, along with the length of the flagella. These morphological and behavioral interstage differences possibly reflect distinct functions in dispersion and invasion of the host and/or prey and may provide novel insight into the virtually unknown function of diplonemids in the oceanic ecosystem.

## INTRODUCTION

Diplonemids constitute a monophyletic group of predominantly deep-sea protists within the phylum Euglenozoa (1) that was recently subdivided into four lineages: the classic diplonemid clade or Diplonemidae, the Hemistasiidae clade, and deep-see pelagic diplonemid (DSPD) clades I and II (2, 3). With over 45,000 operational taxonomic units (OTUs), diplonemids have been recently characterized as the most species-rich group of marine planktonic eukaryotes (2, 4, 5). Despite their enormous diversity and abundance in the ocean, they remain one of the most poorly studied groups of protists. Indeed, a century after the first report on diplonemids was published (6), there are only nine species for which both sequence data and comprehensive morphological description are available (7). Most representatives of the DSPD I clade, which accounts for 97% of all diplonemid diversity (2), are known only as region V9 of the 18S rRNA gene, although the first information about their morphology and genome has been recently reported (3, 8).

To fill this gap in our knowledge, we launched a project in which diplonemids were screened in coastal surface and deep-sea waters around Japan. We obtained two isolates that fall within the classic diplonemid clade and represent new species. Until recently, the *Diplonemidae* clade consisted of only two genera, *Diplonema* and *Rhynchopus*, whereas Hemistasiidae contained a single species, *Hemistasia phaeocysticola*. On the basis of 18S rRNA phylogeny, the genus *Diplonema* is currently composed of two described species, *D. ambulator* (9, 10) and *D. papillatum* (11, 12), not yet formally described *Diplonema* sp. strains ATCC 50225 and ATCC 50232, and a number of environmental sequences unavailable in culture (7). Traditionally, diplonemids that possess a nonflagellated stage within their life cycle were classified into the genus *Rhynchopus*, whereas all those with permanently protruding flagella were considered members of the genus *Diplonema* (13, 14).

In this work, we also describe the first case of bacterial endosymbiosis in diplonemids. Endosymbiotic relationships have evolved multiple times independently in a number of unrelated groups of protists (15, 16). By far the most frequently reported protist taxa involved in symbiotic relationships with bacteria are aerobic ciliates and amoebas (15, 17–20), while their anaerobic counterparts establish symbioses with both archaea and bacteria (21–23). Reports on endosymbioses in Euglenozoa are rather scarce, although several cases have been described in three major groups, namely, kinetoplastids (24–26), euglenids (27–29), and possibly symbiontids (30). Diplonemids prominently remained the only group within Euglenozoa with no record of endosymbiotic bacteria. Here we report the presence of *Holospora*-like symbionts that reside within the novel isolates and display a unique interaction with the host cell as they occupy both the cytoplasm and the mitochondrion.

## RESULTS

### Molecular phylogenetic position of diplonemids.

**FIG 1:**
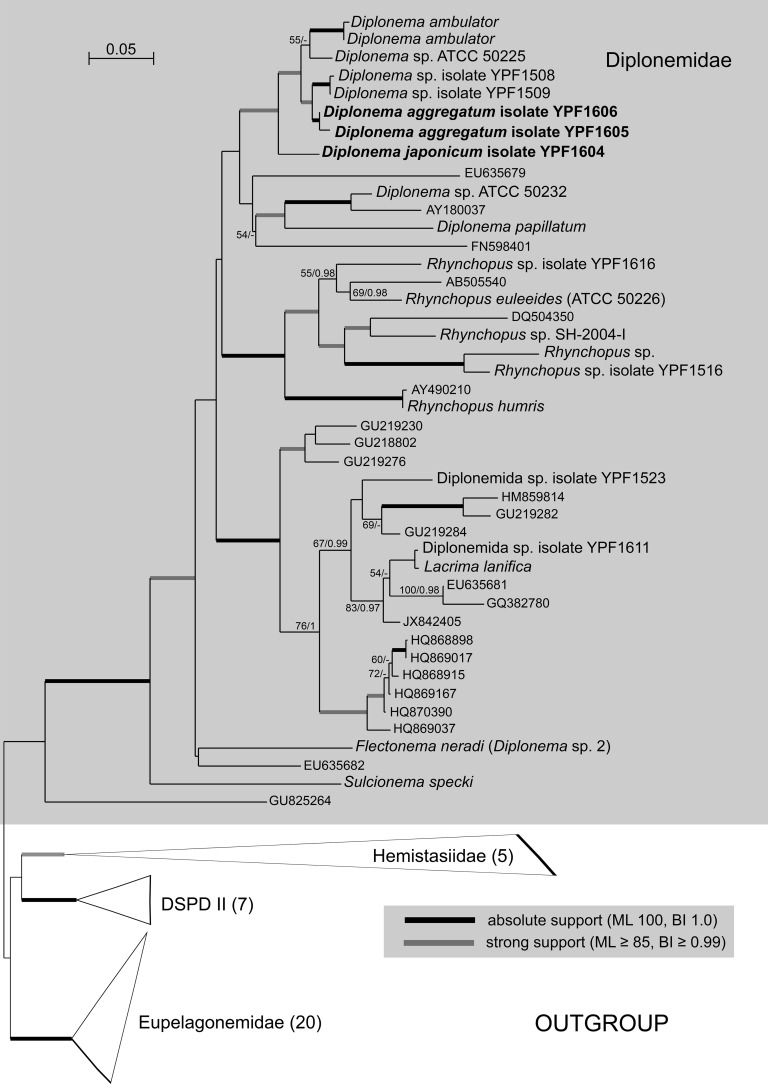
Maximum-likelihood (ML) phylogeny of novel diplonemid endosymbiont-containing species (in bold font) based on an 18S rRNA data set (93 taxa, 1,909 alignment positions) inferred in RAxML 8.2.4 under the gamma-corrected GTR model. Branching support (values at respective nodes) is represented by nonparametric bootstrapping (BS) estimated from 1,000 replicates by a thorough algorithm, as well as by Bayesian posterior probabilities (PP) estimated in Phylobayes 4.1 (C40+GTR model). For Bayesian inference (BI), 0.95 probability was used as a support criterion. See respective parts of Text S1 for details of the phylogenetic analyses. Bold lines represent absolute support (100 BS/1.0 PP).

Both maximum-likelihood and Bayesian inference analyses of an 18S rRNA gene data set with exhaustive sampling of *Diplonemidae* confirmed the placement of the newly described species within the *Diplonema* clade. Representatives of this genus are split into two lineages; one consists of *D. papillatum*, *Diplonema* sp. strain ATCC 50232, and three uncultured diplonemids, while the second, well-supported, clade contains *D*. *ambulator*, *Diplonema* sp. strain ATCC 50255, *Diplonema aggregatum* sp. nov., and *Diplonema japonicum* sp. nov. (see below), the most basal member of this group. The 18S rRNA genes of the two species (accession no. MF422190 and MF422192) differ from each other by 93 nucleotides, which translates into 95.5% sequence identity. Both phylogenetic position and sequence divergence qualify them as new diplonemid species (Fig. 1).

10.1128/mBio.02447-17.1 TEXT S1  Supplemental methods used in this study, Download TEXT S1, DOCX file, 0.02 MB. Copyright © 2018 Tashyreva et al. 2018 Tashyreva et al. This content is distributed under the terms of the Creative Commons Attribution 4.0 International license.

### Light microscopy and cell cycle.

Isolates YPF1603 and YPF1604 (holotype) are named *Diplonema japonicum* sp. nov. here.

**FIG 2:**
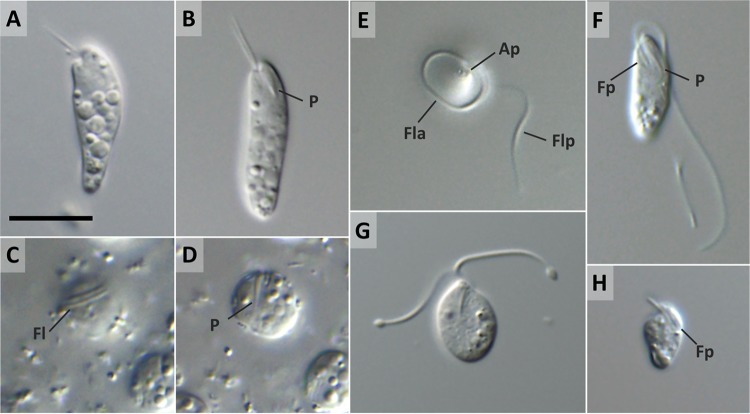
Differential interference contrast micrographs of *D. japonicum* sp. nov. (A, B) Trophic cells with a widened anterior half tapering posteriorly (A) or showing a symmetrical elongated shape (B); note the prominent cytopharynx (P). (C, D) Solitary sessile cell focused on flagella (Fl) coiling around the cell apex (C) and on the cytopharynx (D). (E, F) Swimming cell with the anterior flagellum (Fla) in the form of a loop around the anterior half and the posterior flagellum (Flp) extended (E) and cell with both flagella extended (F); note the pronounced apical papilla (Ap), flagellar pocket (Fp), and cytopharynx (P). (G) Slightly compressed swimming cell with notably swollen flagellar tips during flagellar disassembly. (H) Long-term-starved miniature cell with short flagella. Scale bar is 10 μm.

Trophic cells in well-growing cultures are cylindrical, elongated, and tapered posteriorly, with a wide anterior half that is slightly constricted and bent at the cell apex (Fig. 2A). Less commonly, trophic cells have a nearly symmetrical elongated shape with both ends rounded (Fig. 2B). The cells measure 15.3 to 22.9 (mean ± SD [standard deviation of mean], 19.9 ± 1.9 [*n* = 25]) μm in length and 4.5 to 7.0 (5.8 ± 0.6) μm in width. Two thin, equally long flagella, about one-third of the cell body in length, are parallel and inserted subapically into a pronounced flagellar pocket (Fig. 2B and F). In fresh cultures, cells mostly glide at the bottom of the culture flasks flexing their flagella, which gives an impression of ambulation; they may also be observed rotating around their anterior part by attaching to the surface via a single flagellum. The gliding cells often drastically change their shape because of metabolic contortions such as contraction-expansion, extension, writhing, and twisting. Numerous refractive vacuoles are spread throughout the cytoplasm, being absent only from the anterior part (Fig. 2A and B). The J-shaped ingestion apparatus, well visible under transmitted light, runs parallel to the longitudinal axis of the cell (Fig. 2B and F).

Within a few hours after placement in starvation medium, virtually all cells round up and attach firmly to the bottom of the culture flask (Fig. 2C and D). Closer examination reveals that they are not round but folded up and enclosed in a translucent mucilaginous coat through which they appear to attach to the surface. The sessile cells are solitary, measure 6.5 to 8.5 μm in diameter, turn slowly within the mucilaginous envelope, and produce long flagella that wrap around the anterior end (Fig. 2C and D). Upon further extension of starvation by 24 h, the fully flagellated stage begins to detach from the bottom of the flask. It is 10.3 to 12.7 (12.7 ± 1.0 [*n* = 10]) μm long and 4.0 to 5.2 (4.6 ± 0.4) μm wide and oval or tapered posteriorly and generally lacks refractive vacuoles (Fig. 2F). Swimming is mediated by two thick, unequal flagella usually at least twice as long as the cell (Fig. 2F). The posterior flagellum is loosely twisted around the cell and stretches behind it, while the anterior flagellum forms a tight loop around the anterior half (Fig. 2E). The swimming cells are fast and move in a straight line continuously rotating around their longitudinal axis. Metabolic movements are also typical for this stage, especially when cells are immobilized under a coverslip. The actively swimming phase is transient, usually lasting less than 12 h. Further starvation leads to miniaturization of the cells and flagellar disassembly, accompanied by pronounced swelling of the flagellar tips (Fig. 2G). During this terminal stage of starvation, cells are incapable of movement; possess very short, thin flagella; and reduce their body size to 7.0 by 3.5 μm (Fig. 2H). Eventually, within several days of starvation, the cells undergo lysis. However, if placed in fresh medium, they readily revert to the trophic stage. While the starved cells pass transitions between phases nearly synchronously, old batch cultures exhibit a mixture of trophic, sessile, fast-swimming, and miniaturized cells.

Isolates YPF1605 and YPF1606 (holotype) are named *Diplonema aggregatum* sp. nov. here.

**FIG 3:**
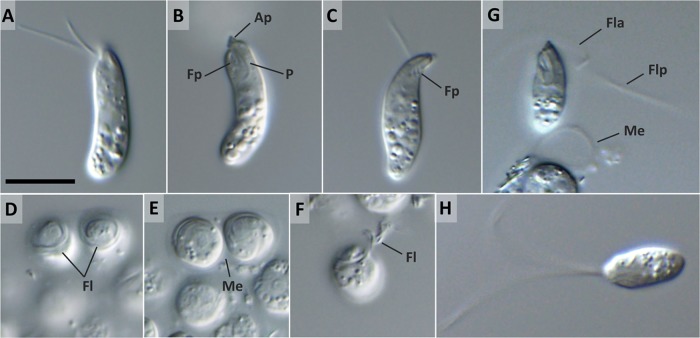
Differential interference contrast micrographs of *D. aggregatum* sp. nov. (A to C) Trophic cells with an elongated shape (A), a C shape (B), and an S shape (C); note the prominent apical papilla (Ap), flagellar pocket (Fp), and cytopharynx (P). (D, E) Group of sessile cells from old batch cultures with flagella (Fl) coiled around the anterior end (D) and enclosed in a mucilaginous envelope (Me) involved in surface attachment (E). (F) Sessile cell with partially unwound flagella (Fl) in transition to the swimming stage. (G) Recently emerged swimming cell with the anterior flagellum (Fla) forming a loop around the anterior half and an extended posterior flagellum (Flp); note the empty mucilaginous envelope (Me) remaining after release of the cell. (H) Swimming cell with fully extended flagella. Scale bar is 10 μm.

Notably elongated, flattened trophic cells with acute anterior and round posterior ends constitute the predominant morphotype in fresh culture (Fig. 3A), although slightly C-shaped (Fig. 3B) or S-shaped (Fig. 3C) cells are also frequently seen. The characteristic apical papilla and ingestion apparatus are well visible by light microscopy (Fig. 3B), as are large refractive vesicles in the posterior half. The cell length ranges from 17.6 to 24.7 (21.6 ± 1.9 [*n* = 25]) μm, and the width is between 4.3 and 6.2 (5.2 ± 0.54) μm. Two parallel-oriented and unequally long, thin flagella emerge from a deep subapical flagellar pocket (Fig. 3B and C). The longer flagellum measures approximately half of the body length and is often twice as long as the shorter flagellum (Fig. 3A). The cells move by gliding, which is accompanied by slow ambulation by flexing of both flagella. Contracting-extending and twisting metabolic movements are frequent among the gliding trophic cells.

In old batch cultures, the cells round up, cluster, and attach firmly to the flask bottom to form extensive, often macroscopic aggregates. However, such sessile cells are never produced by starvation of the trophic cells in seawater. Rounded cells are 6.5 to 8.5 μm in diameter and embedded in a conspicuous amount of a mucilaginous substance that glues neighboring cells together and attaches them to the flask (Fig. 3D and E). Like those of *D. japonicum*, sessile cells remain active and synthesize long, thick flagella coiling around themselves (Fig. 3D and F). The transition from the sessile stage to free-swimming flagellates does not occur following prolonged starvation in either old culture medium or seawater. The release of swimming stages requires incubation of the sessile cells in seawater, followed by the addition of a small portion of LB agar. The mucilaginous envelopes produced by the sessile stage persist upon the emergence of trophic or swimming cells (Fig. 3G). While the morphology and behavior of the swimming stage of *D. aggregatum* are indistinguishable from those of *D. japonicum* (Fig. 3G and H), this phase is much shorter in the former, lasting only a couple of hours. Upon the release of nutrients from the LB agar, the cells revert to the trophic stage. When placed in seawater, swimming cells quickly reduce the size and length of their flagella to produce miniaturized trophic cells, which are incapable of movement and lack cytoplasmic refractive inclusions (data not shown).

### Morphology and localization of bacterial endosymbionts.

**FIG 4:**
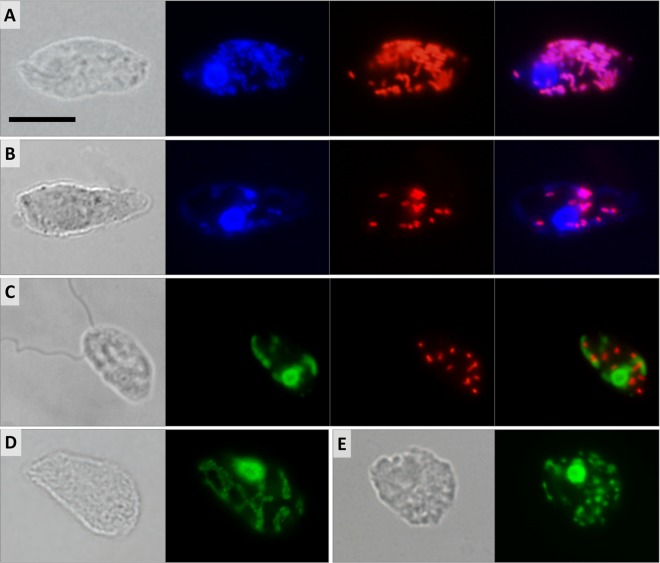
Differential interference contrast and fluorescence micrographs of *D. japonicum*. (A, B) Trophic cell with numerous (A) and few (B) cytoplasmic bacteria. Shown from left to right are a differential interference contrast image, DAPI staining as three overlaid images focused through the cell, FISH probe fluorescence, and overlaid DAPI and FISH images. (C) Swimming cell. Shown from left to right are a differential interference contrast image, SYTO24 staining as two overlaid images, FISH probe fluorescence, and overlaid SYTO24-stained and FISH images. (D, E) Differential interference contrast and SYTO24-stained images of trophic cells. Shown is selective staining of the host nuclear and mitochondrial DNA forming a net (D) or splitting into small patches (E). Scale bar is 10 μm.

**FIG 5:**
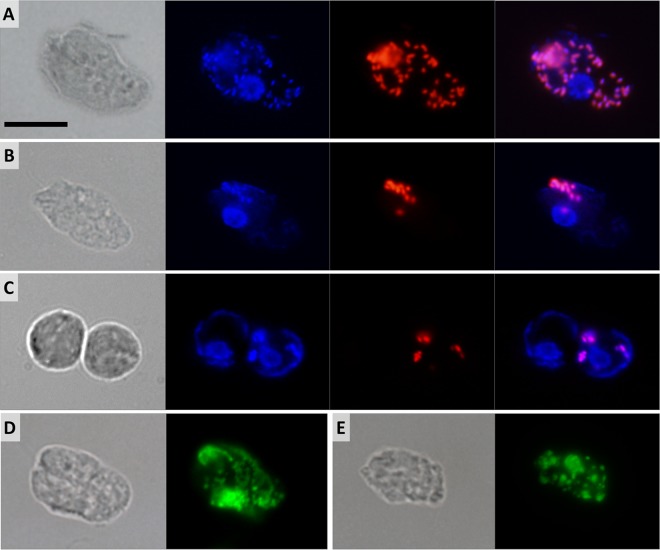
Differential interference contrast and fluorescence micrographs of *D. aggregatum*. (A, B) Trophic cells with numerous (A) and few (B) cytoplasmic bacteria. Shown from left to right are a differential interference contrast image, DAPI staining as two overlaid images focused through the cell, FISH probe fluorescence, and overlaid DAPI and FISH images. (C) Sessile cells. Shown from left to right are a differential interference contrast image, DAPI staining as two overlaid images, FISH probe fluorescence, and overlaid DAPI and FISH images. (D, E) Differential interference contrast and SYTO24-stained images of trophic cells. Selective staining of the host nuclear and mitochondrial DNA forming a net (D) or splitting into small patches (E). Scale bar is 10 μm.

Bacterial cells were periodically seen in axenic cultures of *D. japonicum* and *D. aggregatum* following lysis of the protists. Hybridization with the EUB338 probe, which targets most bacterial groups, revealed numerous rod-shaped cells scattered throughout the host cytoplasm (Fig. 4A and 5A). The signal was never detected in nuclei or extracellularly. All trophic cells of both diplonemids were positive, with the number of bacteria ranging from <10 (Fig. 4A and 5A) to >100 (Fig. 4B and 5B) per host cell.

The endosymbionts in both species are short rods 1.0 to 1.4 μm long and 0.5 to 0.6 μm thick. All sessile and swimming *D. japonicum* cells contain cytoplasmic bacteria, although often at lower counts (Fig. 4C). In contrast, sessile and swimming *D. aggregatum* cells usually bear very few bacteria per cell, often arranged in small clusters or lacking any bacteria at all (Fig. 5C). Following 2 weeks of starvation, not only does the number of endosymbionts drop, but the fluorescence intensity produced by the EUB338 probe is reduced, possibly reflecting a decrease in the number of ribosomes in bacteria within the starved host (data not shown). The morphology of the endosymbionts remains unaltered throughout the host cell cycle, with no signs of differentiation into long infectious forms, which are common in *Holospora*-like bacteria (31). Antibiotic treatment proved unsuccessful at removing endosymbionts; however, incubation with kanamycin transiently reduced the number of bacteria to 10 per *D. aggregatum* cell (data not shown).

### Fluorescence staining of organelles.

Multiple attempts to label the mitochondrion with specific membrane potential-based probes and immunofluorescence failed in all diplonemid stages. However, we were able to visualize the single reticulated mitochondrion by staining its DNA, which is, as in the case of *D. papillatum* (32), extremely abundant and distributed uniformly throughout the organelle. Bacterial nucleoids are brightly stained with 4',6-diamidino-2-phenylindole (DAPI) and precisely follow the pattern of the EUB338 probe fluorescence (Fig. 4A and B and 5A and B). In contrast, in both diplonemids, the mitochondrial DNA is stained very poorly with DAPI, which is likely a reflection of its unusually high GC content (33). However, the SYTO24 probe yields very strong fluorescence of the host DNA, both nuclear and mitochondrial, whereas the bacterial nucleoids remain virtually unstained, as SYTO24 fluorescence does not overlap the fluorescence *in situ* hybridization (FISH) signal (Fig. 4C and 5C). The SYTO24 staining suggests that, in both species, the single mitochondrion is a flat, reticulated structure located beneath the cell surface (Fig. 4D and 5D). In some trophic and swimming cells, the mitochondrial DNA seems to be organized into multiple discrete foci (Fig. 4D and 5D). In both species, the nucleus is usually located in the anterior half of the cell, adjacent to the turn of the J-shaped ingestion apparatus (Fig. 4A and B and 5A and B).

### Molecular phylogeny and taxonomy of endosymbionts.

**FIG 6:**
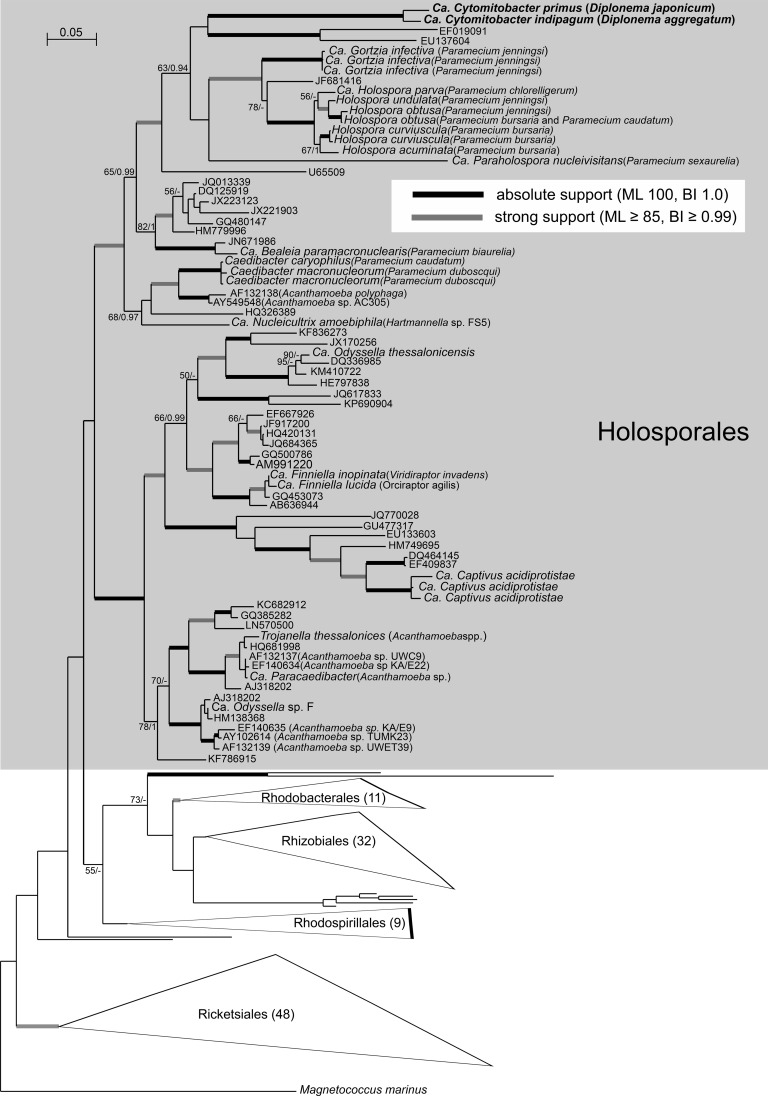
Maximum-likelihood (ML) phylogeny of novel diplonemid-associated bacterial symbionts (in bold font) based on a 16S rRNA data set containing a representative sampling of major alphaproteobacterial lineages with a focus on *Holosporaceae* (179 taxa, 1,390 alignment positions) inferred in RAxML 8.2.4 under the gamma-corrected GTR model. Branching support (values at respective nodes) is represented by nonparametric bootstrapping (BS) estimated from 1,000 replicates by a thorough algorithm, as well as by Bayesian posterior probabilities (PP) estimated in Phylobayes 4.1 (C40+GTR model). For Bayesian inference (BI), 0.95 probability was used as a support criterion. See respective parts of Text S1 for details of the phylogenetic analyses. Bold lines represent absolute support (100 BS/1.0 PP).

Phylogenetic analysis of the 16S rRNA genes of the endosymbionts isolated from *D. japonicum* and *D. aggregatum* confirmed their alphaproteobacterial origin (accession no. MG719947 and MG719948). Both sequences are part of the *Holosporaceae* clade (class *Alphaproteobacteria*, order *Holosporales*, family *Holosporaceae*), which contains bacterial symbionts of ciliates and other protists, such as amoebas, ciliates, and rhizarians. Together with sequences obtained from an aspen rhizosphere (EF019091) and a flea of vertebrates *Oropsylla hirsuta* (EU137604), the endosymbionts of diplonemids form a well-separated clade, sister to all currently recognized candidate holosporaceans, namely, *Holospora*, *Paraholospora*, and *Gortzia* spp. For this novel lineage of bacterial endosymbionts, we have established a new genus, “*Candidatus* Cytomitobacter” (Fig. 6), to accommodate “*Ca*. Cytomitobacter primus” sp. nov. (*D. japonicum*) and “*Ca*. Cytomitobacter indipagum” sp. nov. (*D. aggregatum*).

The observed branching order has a rather low level of support, probably reflecting a general trend of increased rates of evolution typically seen in symbiotic bacteria, which consequently causes problems with their phylogenetic reconstruction. Both of the diplonemid symbionts described here differ in their partial 16S rRNA sequences by 41 nucleotides, which translates into 96% sequence identity. Their distance from other members of the family *Holosporaceae* is, on average, 158 nucleotides in the same gene (85% sequence identity). A sister group of *Holosporales* is the so-called C/N clade, consisting of *Caedibacter* and “*Ca*. Nucleicultrix,” followed by a large assemblage of members of the family “*Ca*. Paracaedibacteraceae,” which is in agreement with current understanding of evolution of symbiotic alphaproteobacteria in protists (34, 35).

### Infection experiments.

Since some *Holospora*-like bacteria are capable of horizontal transmission from one taxonomically related host to another (17), several infection experiments have been performed with the bacteria isolated from diplonemids. However, none of the methods employed to infect several endosymbiont-free diplonemid species were successful. The endosymbionts were incapable of growing in the liquid and agar media tested (data not shown).

### Fine structure.

**FIG 7:**
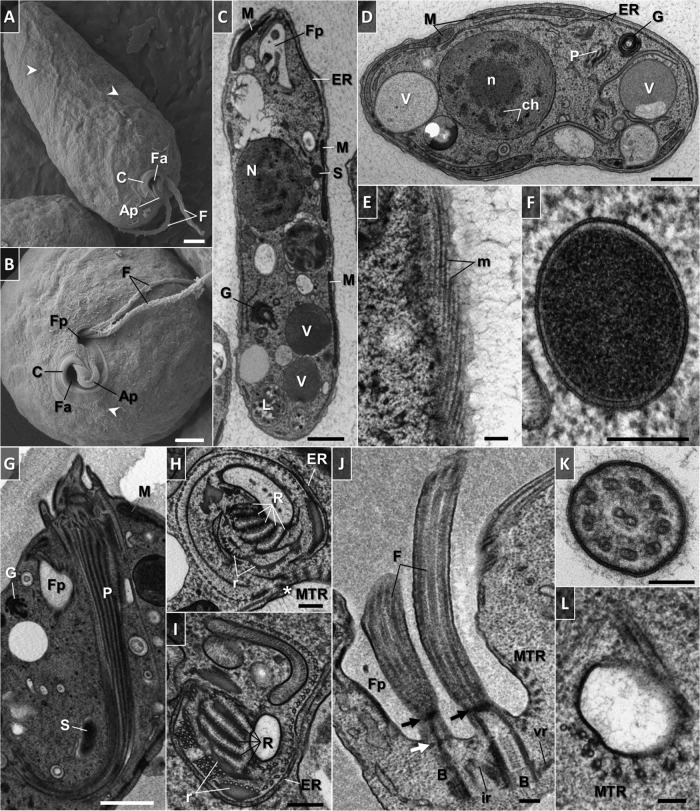
Scanning (A, B) and transmission (C to L) electron micrographs of trophic *D. japonicum*. (A) General appearance of the cell. Note the protruding cytoskeletal microtubules that follow the helix path (arrowheads). (B) Anterior part of the cell. Note the protruding cytoskeletal microtubules near the cytostome (arrowhead). (C) Longitudinally sectioned cell illustrating the main ultrastructural features. (D) Transversely sectioned cell. (E) Tangential section showing an elaborate array of microtubules that follow the helical path along the length of the cytoskeleton. (F) Cross-sectioned endosymbiotic bacterium. Note the double membrane, periplasm, and homogeneous granular interior. (G) Longitudinal profile of a hornlike cytopharynx. The ribs that constitute the cytopharynx appear as a series of longitudinally oriented, electron-dense lines. (H) Cross-sectioned cytopharynx in a region of the cytostome showing a series of five ribs and supporting rods made of clusters of microtubules and fibrous material. Note the row of longitudinally oriented microtubules in close proximity to reinforcing and cytoskeletal microtubules (asterisk). (I) Cross-sectioned proximal end of the cytopharynx. A row of longitudinally oriented microtubules become associated with the rods and ribs. Longitudinally sectioned flagellar pocket. Parallel basal bodies are associated with intermediate and ventral roots. Note the reinforcing microtubules and gap of cytoskeletal microtubules in the flagellar attachment zone. Arrows indicate distal (black) and proximal (white) transitional plates. (K) Transverse section of a flagellum covered with fine hairs. The paraflagellar rod is absent. (L) Cross section through the extension underneath the flagellar pocket, where a band of reinforcing microtubules originates. Abbreviations: Ap, apical papilla; B, basal body; C, cytostome; ch, chromatin; cr, mitochondrial cristae; ER, endoplasmic reticulum; F, flagellum; Fa, feeding apparatus; Fp, flagellar pocket; G, Golgi apparatus; ir, intermediate root; L, phagolysosome-like body; M, mitochondrion; m, microtubules; MTR, reinforcing microtubules; N, nucleus; n, nucleolus; P, cytopharynx; pm, plasma membrane; R, rib; r, supporting rod; S, endosymbiont; V, vacuole; vr, ventral root. Scale bars: 1 µm (A to D, G), 0.2 µm (E, F, H to J), and 0.1 µm (K, L).

**FIG 8:**
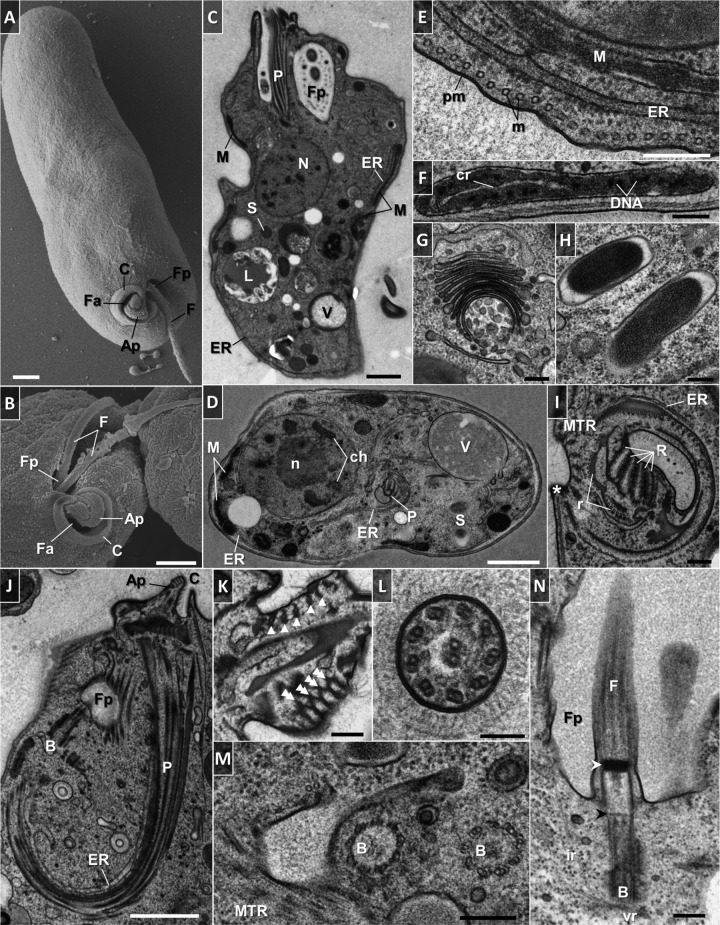
Scanning (A, B) and transmission (C to N) electron microscopy of trophic *D. aggregatum*. (A) General appearance of the cell. (B). Anterior part with feeding apparatus, collar-like cytostome, and subapical flagellar pocket with emerging flagellar joint by the apical papilla. (C) Longitudinally sectioned cell. The cytoplasm is filled with free ribosomes, endosymbionts, reserve granules, vesicular inclusions, and microbodies. (D) Transversely sectioned cell. (E) Cross section showing details of plasma membrane and corset of underlying microtubules. (F) Branch of the mitochondrion with large lamellar cristae having a longitudinal orientation and abundant patches of electron-dense DNA. (G) Golgi apparatus with numerous vesicles and stacked flat cisternae. (H) Rod-shaped bacteria with a homogeneous granular interior and a prominent periplasm. (I) Cytopharynx with five fibrillar ribs arranged in the form of a partial rosette and supported by rods and a row of microtubules. The microtubule row is in close association with reinforcing and cytoskeletal microtubules (asterisk). (J) Longitudinally sectioned feeding apparatus showing the apical papilla and cytostome, which opens into a deep, hornlike cytopharynx. Note the hair coat overlying the surface membrane of the feeding apparatus. (K) Section through the apical papilla showing a bundle of five reinforcing microtubules embedded in fibrous material (arrowheads) that eventually develop into cytopharyngeal vanes (double arrowheads). (L) Cross section of flagellum with fine hairs and lacking the paraflagellar rod. (M) Section through the basal bodies arranged by an assembly of nine microtubular triplets. A structural extension with reinforcing microtubules leading underneath the flagellar pocket is shown. (N) Longitudinal view of the flagellum. Note the distal (white arrowhead) and proximal (black arrowhead) transitional plates, as well as the absence of cytoskeletal microtubules in the region of flagellum attachment. Abbreviations are as in Fig. 7. Scale bars: 1 µm (A to D, J), 0.2 µm (E to I, K, M, N), and 0.1 µm (L).

**FIG 9:**
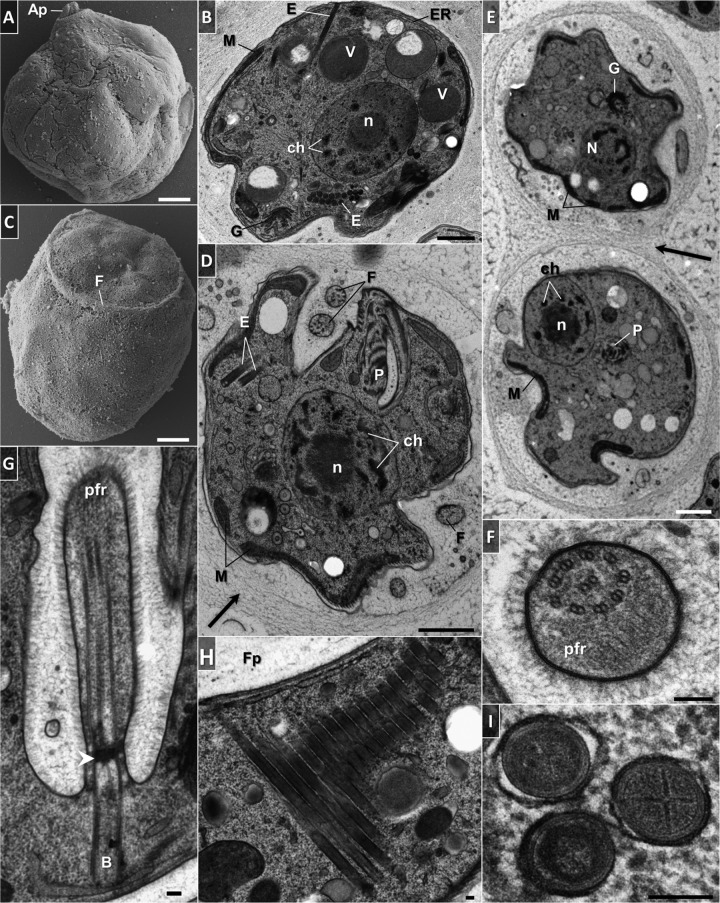
Electron micrographs of sessile *D. japonicum* (A to B, I) and *D. aggregatum* (C to G). (A, C) Morphology of cells exhibiting a globular form with an apical papilla by scanning electron microscopy. (B, D) Ultrastructure of sessile cells. Profiles of the mitochondrial and endoplasmic reticulum networks extend at the cell periphery. Note the extrusomes, single or arranged in a battery, and an amorphous diffuse coat around the cell (arrow). (E) Two sessile cells glued to each other by an amorphous diffuse coat (arrow). (F) Cross-sectioned flagellum with hair and a conspicuous paraflagellar rod. (G) Longitudinal section in the region of flagellar attachment showing an axoneme with a lattice-like paraflagellar rod that joins the axoneme a short distance from the proximal transitional plate (arrowhead). (H) Battery of parallel tubular extrusomes in a longitudinal view. Extrusomes are concentrated around the flagellar pocket. (I) Cross-sectioned tubular extrusomes. Note the cruciate centers and thick membrane. Abbreviations are as in Fig. 7. E, extrusomes. Scale bars: 1 µm (A to E) and 0.1 µm (F to I).

The trophic cells of *D. japonicum* and *D. aggregatum* are smooth with separate feeding and flagellar apparatuses joined by an apical papilla at the anterior end (Fig. 7A and B and 8A and B). The opening to the cytopharynx is represented by a pronounced collar-like cytostome slightly protruding above the cell surface (Fig. 7A and B and 8A and B). When entering the sessile stage, both diplonemids acquire a globular shape with their flagella firmly wrapped around them (Fig. 9A to D). In addition, they become enveloped by an amorphous coat of uniform thickness that seems to help the cells stick to each other (Fig. 9D and E).

**FIG 10:**
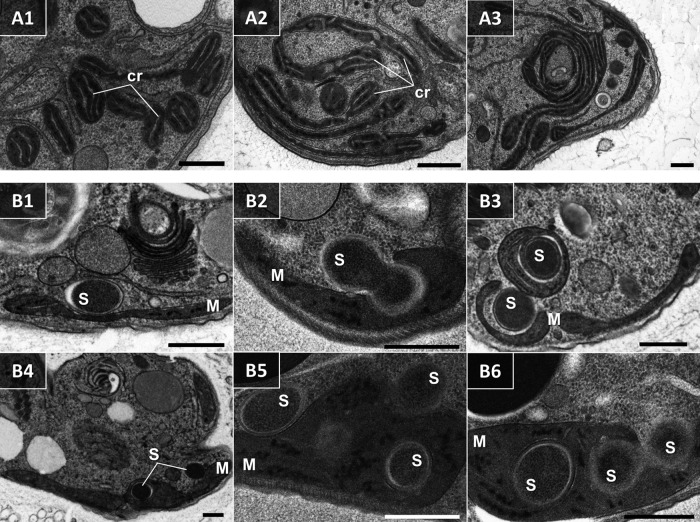
Transmission electron micrographs of *D. japonicum*. (A1 to A3) Nonconsecutive serial sections depicting individual thin mitochondrial branches that form a large, convoluted complex. Mitochondria display characteristic large lamellar cristae (cr) and abundant patches of DNA. (B1 to B6) Sections showing details of the interaction between bacteria (s) and the mitochondrion (m), ending in internalization of the bacterium within the organelle. Scale bars are 0.5 µm.

**FIG 11:**
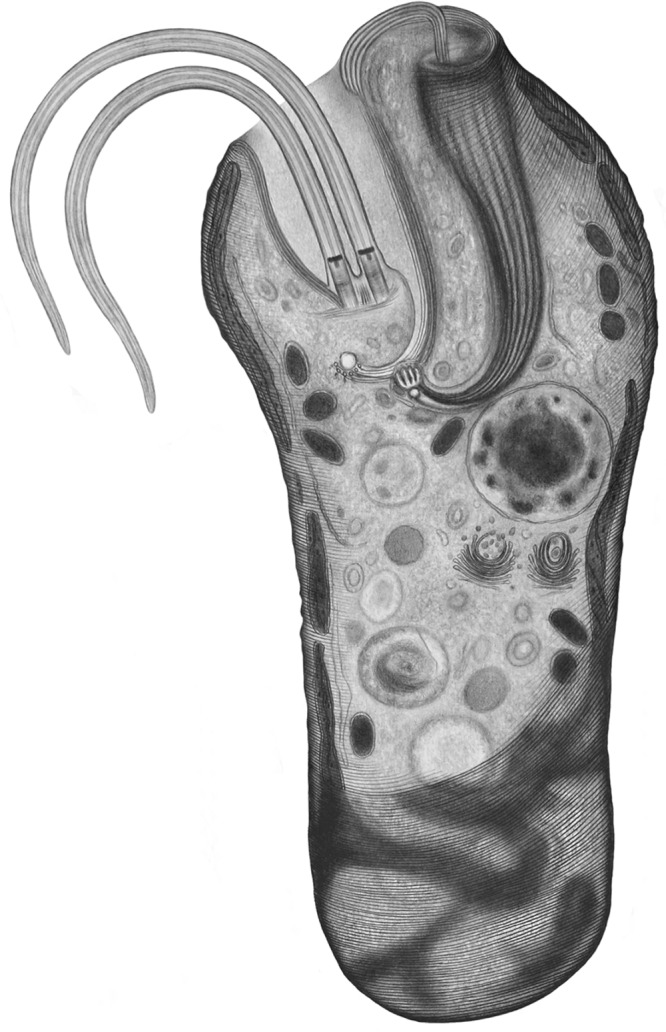
Drawing depicting the general appearance of principal structures within the *D. japonicum* cell at the trophic stage.

The fine structures of the two species are similar (Fig. 7C and D and 8C and D). The cell membrane is supported by a tight corset of cytoskeletal microtubules that enfold the cell longitudinally following an S-helix path (Fig. 7A, B, and E; Fig. 8E; and Fig. 11). The cytoskeletal microtubules are absent from the region of flagellar attachment (Fig. 7J and 8N). Adjacent to them extends a peripheral mitochondrial network that is seen as multiple narrow tubular structures in longitudinal sections (Fig. 7C, D, and G and 8C to F; also see Fig. 11). The only discernible structures in the lumen of the organelle are numerous patches of electron-dense DNA and few large lamellar cristae with a longitudinal orientation (Fig. 8F and 10A1 to A3). Occasionally, individual mitochondrial branches were observed to form complex circling profiles (Fig. 10A1 to A3). Just below and parallel to the mitochondrion, a thin tubular network of endoplasmic reticulum is extended (Fig. 7C and D; 8C to E; and 11), which also tends to be frequently located around the pharynx (Fig. 7D, H, and I and 8D, I, and J). Cells bear a large vesicular nucleus, usually spherical or ellipsoidal, that is situated in the anterior third of the cell (Fig. 7C and D, 8C and D, and 11). It contains one conspicuous nucleolus placed either centrally or eccentrically and a condensed heterochromatin distributed at the periphery (Fig. 7C and D; 8C and D; 9B, D, and E; and 11). Close to the nucleus lies the Golgi complex, which often consists of several distinct bodies, each consisting of 6 to 13 and 4 to 11 prominent, stacked, flat cisternae in *D. japonicum* and *D. aggregatum*, respectively (Fig. 7C, D, and G; 8G, 9B and E; and 11). The posterior part of the cell is filled with various vacuoles with different contents and phagolysosome-like bodies (Fig. 7C and D, 8C and D, and 11). The cytoplasm is packed with free ribosomes, small refractive granules, vesicular inclusions, and microbodies.

A number of rod-shaped intracytoplasmic bacteria are randomly distributed throughout the cell (Fig. 7C and G, 8C and D, and 11). They possess a homogeneously dense granular interior enveloped by single and double membranes with a periplasmic space in between (Fig. 7F and 8H). These bacteria invariably showed no signs of lysis or degradation. They prefer to be localized adjacent to the mitochondrion and were occasionally seen wrapped by the organelle or even establishing themselves within it (Fig. 7C and 11). We were able to reconstruct a series of steps presumably leading to the internalization of the endosymbiont in the mitochondrial lumen (Fig. 10B1 to B6). The interaction seems to be initiated by either penetration of the endosymbiont into the mitochondrion, or active encirclement by the latter (Fig. 10B1 to B6).

In both diplonemids, the feeding apparatus is oriented longitudinally within the cell. It opens with a cytostome that leads into a deep, hornlike, tubular cytopharynx and terminates at the level of the nucleus (Fig. 7D and G, 8D and J, and 11). The cytopharynx is represented by an integrated system of five membranous folds, called vanes or ribs, that originate from the rods made of clusters of microtubules and fibrous material (Fig. 7H and I and 8D and I). This arrangement is structurally supported by a row of longitudinally and transversely oriented microtubules that run on the opposite side (Fig. 7H and 8I). While descending into the cell, the cytopharynx becomes gradually thinner, so that the microtubule row is drawing together with the rods (Fig. 7G and I and 8D and J). The proximal end of the feeding apparatus is often associated with membrane-bounded vesicles (Fig. 7I and 8D). In the cytostomal region, the row of microtubules that lines the cytopharynx occurs in close proximity to the cytoskeletal microtubules, as well as to a complex of reinforcing microtubules (MTR) (Fig. 7H and 8I). A band of MTR originates from a J-like extension underneath the flagellar pocket and stretches along it before reaching the cytostome (Fig. 7L, 8M, and 11). At the cell’s apex, several reinforcing microtubules arch, forming an apical papilla that becomes surrounded by a dense matrix and eventually develops into the cytopharyngeal vanes (Fig. 8K and 11).

Two flagella emanate from parallel basal bodies located at the bottom of a deep flagellar pocket (Fig. 7J and 8N). The basal bodies comprise typical centrioles assembled by nine microtubular triplets (Fig. 8M) and are associated with three asymmetrically arranged microtubular roots (Fig. 7J and 8J and N). One of them is intermediate, while the other two are lateral (also called ventral and dorsal), projecting on the sides of the basal bodies (Fig. 7J and 8N). The axonemes of both flagella are composed of nine outer microtubular doublets and a central pair originating at the distal transitional plate (Fig. 7J and K and 8L and N). Both flagella of trophic cells lack the paraflagellar rod (Fig. 7J and K and 8L and N), while this lattice-like structure is prominently present in flagella of the sessile cells (Fig. 9D, F, and G). The paraflagellar rod joins the axoneme already within the flagellar pocket and spreads along the length of it almost to the tip of the flagellum (Fig. 9G). The apical part of the cell, including the flagellar pocket and both flagella, is covered with a glycocalyx and a hair coat, which appear denser in *D. aggregatum* (Fig. 7K, 8J to L, and 9F and G).

Another distinctive trait of the sessile cells is the presence of tubular extrusomes (Fig. 9B, D, H, and I). They mostly occupy the anterior part of the cell on the side of the flagellar pocket (Fig. 9B, D, and H), though sometimes they may also be irregularly distributed in the posterior part (Fig. 9B). The extrusomes are often arranged in a battery where they are oriented parallel to each other (Fig. 9B and H). These extrusive organelles represent cylinders furnished with cruciate centers that are surrounded by a thick membrane (Fig. 9I).

### Taxonomic summary. (i) Diplonemid hosts.

Phylum Euglenozoa Cavalier-Smith 1981, emend. Simpson 1997; class Diplonemea Cavalier-Smith 1993, emend. Simpson 1997; genus *Diplonema* Griessmann 1913.

### *Diplonema japonicum* sp. nov. Tashyreva, Prokopchuk, and Lukeš (2018) (a) Description.

Species identified by 18S rRNA phylogenetic position. Trophic cells cylindrical, elongated, tapered posteriorly, wide anterior half slightly constricted and bent at cell apex; some with both ends rounded; 15.3 to 22.9 (19.9 ± 1.9) μm long and 4.5 to 7.0 (5.8 ± 0.6) μm wide; numerous refractive vacuoles missing from anterior quarter; flagella equal or subequal third of body length, subapical, regular axonemes, lacking the paraflagellar rod; gliding movement, pronounced metabolic contortions. Starvation-induced sessile stage round, solitary, 6.5 to 8.5 μm in diameter, embedded in mucilaginous coat; long flagella wrapping around anterior end; paraflagellar rods; and tubular extrusomes. Swimming stage emerging from sessile, 10.3 to 12.7 (12.7 ± 1.0) μm long and 4.0 to 5.2 (4.6 ± 0.4) μm wide, oval or tapered posteriorly, lacking refractive vacuoles; swimming fast; unequal flagella twice body length; posterior flagellum stretched; anterior flagellum loops around cell apex, paraflagellar rods, and tubular extrusomes; converted to miniaturized cells measuring 7.0 by 3.5 μm after prolonged starvation. Single reticulated mitochondrion; nucleus anterior.

### (b) Etymology.

Species name is defined by the origin of the isolate.

### *Diplonema aggregatum* sp. nov. Tashyreva, Prokopchuk, and Lukeš (2018) (a) Description.

Trophic cells notably elongated, flattened, acute anterior and round posterior ends, slightly bent into C- or S-shaped cells also observed; large refractive vesicles in posterior half; 17.6 to 24.7 (21.6 ± 1.9 [*n* = 25]) μm long and 4.3 and 6.2 (5.2 ± 0.54) μm wide; two parallel unequally long flagella half of body length, regular axonemes, no paraflagellar rod; gliding on surfaces and metabolic movements. In old batch cultures, sessile cells round, 6.5 to 8.5 μm in diameter, clustered in big aggregates, embedded in mucilaginous substance, long thick flagella around cell apex, a paraflagellar rod, and tubular extrusomes. Swimming stage same as that of *Diplonema japonicum*, released by minor addition of nutrients. Single reticulated mitochondrion; nucleus anterior.

### (b) Etymology.

The species name denotes massive aggregation of cells in the sessile stage.

### Type locality.

The type locality of both species is the Enoshima Aquarium, Kanagawa, Japan.

### Type material.

Hapanthotypes are OsO_4_-fixed slides and ethanol-fixed cells deposited at the protistological collection of the Institute of Parasitology, Biology Center, Czech Academy of Sciences, České Budějovice, no. IPCAS Prot 42 (*D. japonicum*) and 43 (*D. aggregatum*).

### (ii) Endosymbionts.

Class *Alphaproteobacteria* Garrity et al. 2006; subclass *Caulobacteridae* Ferla et al., 2013; order *Holosporales* Szokoli et al., 2016; family *Holosporaceae* Görtz & Schmidt, 2005.

### Genus “*Ca*. Cytomitobacter” gen. nov. Tashyreva, Prokopchuk, and Lukeš (2018).

Genus of endosymbiotic bacteria distinguished from similar genera by molecular phylogenetic analyses. Straight, rod-shaped bacteria 1.0 to 1.4 μm long and 0.5 to 0.6 μm thick, no visible inclusions, spores not observed, Gram-negative cell wall organization. Reside freely in the cytoplasm and occasionally in mitochondria, not surrounded by additional host membranes. Name is derived from the host cell compartments that representatives of the genus inhabit.

### “*Ca*. Cytomitobacter primus” sp. nov. Tashyreva, Prokopchuk, and Lukeš (2018).

Species identified by its unique phylogenetic position on 16S rRNA gene tree. Type host *Diplonema japonicum*. Name (*primus*) denotes it as first described species of the genus.

### “*Ca*. Cytomitobacter indipagum” sp. nov. Tashyreva, Prokopchuk, and Lukeš (2018).

Species identified by its unique phylogenetic position on 16S rRNA gene tree. Type host *Diplonema aggregatum*. Name (*indipagum*) represents abbreviation of the phrase “in *Diplonema aggregatum*.”

## DISCUSSION

### Phylogeny of diplonemids.

The novel diplonemid species described here are robustly placed within the family *Diplonemidae* close to *D*. *ambulator*, thus clearly belonging to the genus *Diplonema*. The low support for the whole *Diplonema* clade, as well as the relatively short branch separating it from the *Rhynchopus* lineage, indicates the lack of molecular synapomorphies defining the current concept of the genus *Diplonema* and the limits of resolution provided by the 18S rRNA gene. Further detailed comparative study, including phylogenomics along with ultrastructural and life cycle data, will hopefully shed more light on the status of this genus. All euglenozoan apomorphies, such as the tripartite flagellar root complex, the lattice-like paraflagellar rod, and the thick-walled tubular extrusomes (14, 36), are present in both newly described *Diplonema* species. Moreover, they share a set of morphological traits with other diplonemids, such as a prominent apical papilla, a tubular ingestion apparatus contiguous to a deep flagellar pocket, two parallel flagella, metaboly, and a tightly packed microtubular corset (7).

All of the diplonemids studied so far have a relatively uniform ultrastructural organization (11, 37), with the distinguishing features, such as size and number of vacuoles, presence of extrusomes and a paraflagellar rod, length of flagella, body shape and size, and the character of movement, being subject to alteration and variability under different conditions and/or in different life cycle stages (7, 14, 38; this work). Moreover, distantly related species may exhibit similar morphology, as in the case of *Flectonema neradi*, which closely resembles representatives of the *D. ambulator* clade and was originally deposited in the American Type Culture Collection as *Diplonema* sp. yet is rather distantly related to it according to the 18S rRNA-based phylogeny (7) (Fig. 1). Therefore, the establishment of the new taxa presented here is based primarily on their unique 18S rRNA gene sequences, although their morphology is sufficiently different from that of other *Diplonema* species for which only morphological data are available (see Table S1 in the supplemental material).

10.1128/mBio.02447-17.2 TABLE S1  Comparison of morphology, ultrastructure, and movement of described species of the genus *Diplonema*. Download TABLE S1, DOCX file, 0.02 MB. Copyright © 2018 Tashyreva et al. 2018 Tashyreva et al. This content is distributed under the terms of the Creative Commons Attribution 4.0 International license.

Some unique characteristics of *D. aggregatum* and *D. japonicum* are worth further attention, as so far they have not been described in this group of protists. Both species form a morphologically distinct trophic stage in the nutrient-rich environment and a sessile stage when nutrients are limited in which long paraflagellar rod-bearing flagella and extrusomes are produced, and finally, a highly motile swimming stage is released upon further starvation or addition of small amount of nutrients. We know close to nothing about the ecologic roles of diplonemids in the ocean, yet the observed morphological alterations may point to (significantly) different strategies of individual stages in the life cycle. Specifically, the trophic stage may possibly be maintained inside the host or prey and, upon death or the exhaustion of nutrients, undergo transformation via the sessile stage into the swimming stage, which seems to be well equipped for dissemination and active searching for a host or prey. Although the role of tubular extrusomes has not yet been investigated, we hypothesize that they may play a role in the invasion process. The presence of batteries of tubular extrusomes in the apical part of *H. phaeocysticola*, the only diplonemid for which active predation has been described so far (39), supports this notion.

*D. aggregatum* and *D. japonicum* are the first members of the genus *Diplonema* in which a swimming stage was described (Table S1). Remarkably, cells in the trophic stage bear thin flagella with a regular axoneme arrangement lacking the paraflagellar rod, while this prominent structure supports flagella of the swimming stage. So far, all of the other ultrastructurally studied members of the genus *Diplonema*, namely, *D. nigricans* (40), *D. ambulator* (37), and *D. papillatum* (11), lack the paraflagellar rod in their trophic stage, and the same applies to *Rhynchopus* spp. (7, 13, 41). However, unlike the *Diplonema* species, members of the genus *Rhynchopus* carry only flagellar stubs with largely disordered axonemes that are concealed inside their flagellar pockets (14), while all of the remaining diplonemid genera, namely, *Flectonema*, *Lacrimella*, and *Sulcionema*, bear this prominent flagellar structure in their trophic stage (7). Therefore, we suggest that the absence of the paraflagellar rod in both flagella in the trophic stage and its emergence in the swimming stage may provisionally serve as an apomorphy for the genus *Diplonema*.

Our finding that in *D. japonicum* and *D. aggregatum*, the trophic stage lacks the paraflagellar rod while the swimming stage builds it is significant. While this well-known feature of the euglenozoan flagellum can be greatly reduced in some kinetoplastid flagellates (42), it is considered to be a canonical organelle, at least in kinetoplastids (43). At this point, we cannot exclude the possibility that the paraflagellar rod, which is composed of dozens of specialized proteins (43), is suppressed yet still present in the trophic stage. However, the observed interstage difference in the cross-sectioned flagellum of both diplonemid species is unprecedented and worth further investigation, as it implies that distinct stages of one species may build a dramatically different flagellum. Since *D. papillatum* was recently turned into a genetically tractable organism (44) and since different stages can be obtained in culture, the observed differences in flagellar structure can be studied by methods of functional genomics.

### Phylogeny of endosymbionts and their interaction with diplonemid hosts.

Phylogenetic analyses revealed that both new bacteria are members of family *Holosporaceae*, which mostly contains other endosymbionts of protists. The host repertoire of this alphaproteobacterial clade, so far containing ciliates, amoebas, and rhizarians, is now expanded to accommodate the endosymbionts of euglenozoans described here. The position of “*Ca*. Cytomitobacter primus” and “*Ca*. Cytomitobacter indipagum” within the order *Holosporales*, investigated by maximum-likelihood analysis (under the standard gamma-corrected GTR model) and the empirical admixture model (GTR+CAT40) employed in Bayesian inference analysis, is not robustly supported, and both endosymbionts stand out from the core representatives of *Holosporaceae* because of their highly divergent 16S rRNA sequence. While the observed topology may be an artifact caused by heterotachy, we tried to overcome the high evolutionary rates and consequent problems with phylogenetic reconstruction by using robust admixture CAT models. We therefore believe that the observed topology reflects the true state and the candidate bacterial species “*Ca*. Cytomitobacter primus” and “*Ca*. Cytomitobacter indipagum” form a new genus within the family *Holosporaceae*.

*D. japonicum* and *D. aggregatum* are closely related, and the same holds true also for “*Ca*. Cytomitobacter primus” and “*Ca*. Cytomitobacter indipagum” (Fig. 1 and 6). In fact, sequences of the endosymbionts are more similar to each other than those of their diplonemid hosts are to each other. Although this difference is only minor, it is in contrast to the commonly accepted scenario of accelerated evolutionary rates in organisms with the parasitic and/or symbiotic lifestyle (45, 46). This discrepancy brings us to the question of the origin of bacterial endosymbiosis in diplonemids. The most parsimonious explanation of the observed relationships is cospeciation of diplonemid hosts and their bacteria. If this is true, the phylogeny of the hosts would be mirrored by the phylogeny of their endosymbionts. However, while both bacterial sequences are monophyletic, this is not the case for the corresponding diplonemid hosts. *D. japonicum* is the most basal representative of a clade containing *D. aggregatum* and the other novel diplonemid isolates YPF1508 and YPF1509, as well as *D. papillatum* and *Diplonema* sp. strain ATCC 50232. Out of these, to the best of our knowledge, only *D. japonicum* and *D. aggregatum* contain bacterial endosymbionts.

This discrepancy can be explained by two alternative scenarios of endosymbiont acquisition in the genus *Diplonema*. The first one postulates that the phylogeny of the endosymbionts follows that of their hosts, with symbiosis being lost in most members of the *Diplonema* clade. The 16S rRNA gene of the endosymbionts is under selection, and thus, evolutionary rates (at least of this gene) are rather low. The second scenario supposes a single origin of endosymbiosis in either of the diplonemid species, followed by horizontal transfer of the bacterium to the other diplonemid host. If this is correct, these diplonemid species would have to have been in close contact at least for some time in their evolutionary history. Interestingly, both *D. japonicum* and *D. aggregatum* were isolated during a single sampling event from the same spot, so such contact is possible. At the current state of knowledge, we slightly prefer the second scenario; however, a detailed genomic survey of both the hosts and endosymbionts is necessary to discriminate between these two hypotheses.

The representatives of *Holosporales* are very common endosymbionts of amoebas and ciliates, in which they colonize various cell compartments, including the cytoplasm and micro- and macronucleus (17, 20, 47, 48). In fact, this group of bacteria consists exclusively of obligate endosymbionts of a variety of protist hosts (49). The role of these bacteria is mostly unknown (17, 18), although the available data suggest that the interactions of *Holosporales* with protists are complex. They may become beneficial or disadvantageous to their hosts under specific conditions or at certain life stages. For instance, endosymbionts reduce the ciliate growth rate but, at the same time, provide a competitive advantage to their hosts over taxonomically related species by releasing killer toxins (50). *Holospora*-like bacteria may endow their host with a tolerance to heat shock (51), whereas other species induce host cell lysis at elevated temperatures (47). As far as we know, no mutual exchange of nutrients and metabolic complementation has been reported for the members of *Holosporales*.

In the case of *D. japonicum* and *D. aggregatum*, understanding of the significance their endosymbionts was impeded by the failure to eliminate them, which we explain by the possible resistance of these bacteria to a variety of antibiotics and/or failure of the drugs to penetrate the host plasma membrane. Nevertheless, we hypothesize that under the conditions studied, the endosymbionts do not negatively affect their host, as they grow well and lyse only in old batch cultures or upon long-term starvation. Moreover, the endosymbiosis appears to be stable and species specific, without differentiation of bacteria into infectious and reproductive forms, a common behavior of bacteria of the genus *Holospora* (17). Better understanding of this endosymbiotic relationship will require sequencing and annotation of the endosymbionts’ genomes, as well as further attempts to eliminate the bacteria via a wider spectrum of antibiotics and cell sorting. In any case, the presence of endosymbionts may help explain the recently described extreme ecologic flexibility of diplonemids, which seem to be omnipresent in the world’s oceans (2, 4, 5, 8).

The endosymbiotic bacteria are found inside mitochondria extremely rarely. In fact, only a small proportion of endosymbiont-infected mitochondria were described in ciliates (52), while the only documented case of bacteria residing predominantly within the organelle is a *Rickettsia*-like organism, “*Ca*. Midichloria mitochondrii,” an endosymbiont found in the ovaries of female *Ixodes ricinus* ticks (53). Remarkably, the massive multiplication of bacteria within the mitochondria and consumption of the organellar content do not disturb the normal development of eggs in the tick host. In a situation reminiscent of the ciliates, we only occasionally detected bacteria inside the mitochondrial matrix. However, we were able to map a sequence by which the bacterium establishes itself inside the organelle. Thanks to its availability in culture, this unique endosymbiont-host system is amenable to detailed dissection in future studies.

## MATERIALS AND METHODS

Axenic clonal cultures were established from samples of seawater by manual cell picking, followed by treatment with antibiotics and cultivation in horse serum-containing liquid medium. The presence of endosymbionts was verified by FISH with a 5′ Cy3-labeled EUB338 probe. DNA was stained with DAPI or SYTO24. For visualization of mitochondria, samples were treated with the membrane potential-sensitive dye tetramethylrhodamine ethyl ester, DiOC_6_(3), MitoTracker Green FM, or MitoTracker Red CMXRos (Life Technol). An immunofluorescence assay targeting mitochondrial heat shock protein 70 (HSP70) was performed with antibodies generated against *Trypanosoma brucei* HSP70. For elimination of bacteria, the culture was treated with the antibiotic chloramphenicol, ampicillin, kanamycin, gentamicin, or azithromycin or a penicillin-streptomycin-neomycin cocktail. Infection experiments were carried out by coculturing of endosymbiont-free diplonemid species with endosymbiont-bearing *D. japonicum* and *D. aggregatum* or by incubation with their cell lysates. For a detailed description of the procedures, as well as DNA isolation, amplification, phylogenetic analyses, and light and electron microscopy, see Text S1.

### Accession number(s).

The GenBank accession numbers of the sequences described here are MF422190 (*D. japonicum*), MF422192 (*D. aggregatum*), MG719947 (“*Ca*. Cytomitobacter primus”), and MG719948 (“*Ca*. Cytomitobacter indipagum”).
