# Supplementary material for: Life Cycle, Ultrastructure, and Phylogeny of New Diplonemids and Their Endosymbiotic Bacteria
Source: mBio. 2018 Mar 6;9(2):e02447-17. doi: 10.1128/mBio.02447-17 (PMC5845003; doi:10.1128/mBio.02447-17)
Supplement: TEXT S1 [file mbo001183766s1.docx]

**SUPPLEMENTARY MATERIALS AND METHODS**

**Isolation and cultivation.** Material from sand filters of Enoshima Aquarium tanks (Kanagawa, Japan) was inoculated into a seawater-based Hemi medium (7), containing a 10 μl∙ml^-1^ antibiotic cocktail (P4083, Sigma-Aldrich). Diplonemid-like cells were isolated under the inverted microscope CKX31 (Olympus) with glass microcapillaries. The axenic clonal cultures were established from a single cell, and subsequently incubated in Hemi medium without antibiotics at 13 to 15°C. For some experiments, the cultures were starved in seawater.

**Fluorescence *in situ* hybridization (FISH) and DNA staining.** Cells were fixed with 4% paraformaldehyde (pFA) or 2% OsO_4_ seawater-based solutions for 20 min, washed off the fixative and re-suspended in distilled water (dH_2_O). Cell suspension was applied on poly-*L*-lysine-coated glass slides and air-dried. Adhered cells were de-hydrated with 50, 80 and 96% ethanol solutions for 3 min. The slides were treated with a hybridization buffer (900 mM NaCl, 20 mM Tris/HCl, 0.01% SDS) containing 35% (v/v) formamide and 5 ng∙μl^-1^ EUB338 probe (5'-GCTGCCTCCCGTAGGAGT-3') labelled with 5'-Cy3 fluorescent dye. The samples were incubated in humidity chambers at 46°C for 90 min, followed by incubation in washing buffer (80 mM NaCl, 20 mM Tris/HCl, 0.01% SDS) on a shaker at 48°C for 30 min. Finally, the slides were rinsed with dH_2_O and air-dried. Freshly fixed or FISH-labelled samples were incubated in 1 μM SYTO24 water solution (Life Technol.) for 20 min, thoroughly rinsed with dH_2_O, air-dried and mounted in ProLong Gold antifade reagent (Life Technol.). Alternatively, fixed samples were directly mounted in ProLong Gold antifade reagent (Life Technol.) containing 4',6-diamidino-2-phenylindole (DAPI).

**Visualization of mitochondria**. Live cells were pelleted by centrifugations, re-suspended in seawater and treated for 30 min with (i) 60 nM tetramethylrhodamine ethyl ester (TMRE), (ii) 2, 6, 10 or 20 μM DiOC_6_(3), (iii) 100 or 200 nM MitoTracker Green FM, and (iv) 100 or 500 nM MitoTracker Red CMXRos (Life Technol.). Immunofluorescence assay (IFA) targeting mitochondrial heat shock protein (HSP) 70 was performed following the protocol by Zíková et al. (54), using antibodies generated against *Trypanosoma brucei* HSP70 (55). The slides were counterstained with DAPI or SYTO24 and prepared as described above.

**Light and fluorescence microscopy.** Light microscopy was done using Olympus BX53 equipped with differential interference contrast (DIC). All slides labelled with fluorescent dyes were observed with the AxioPlan 2 fluorescence microscope (Carl Zeiss Microscopy GmbH, Jena, Germany). For live imaging, a small aliquot was placed between a slide and a cover glass, and the edges were sealed with nail polish. Images and videos were captured with a DP72 microscope digital camera at 1600×1200-pixel resolution using CellSens software v. 1.11 (Olympus). For cell measurements, the images of cells in culture flasks were taken using Zeiss Axio Vert inverted microscope with PlasDIC contrast. The images were processed using GIMP v. 2.8.14, Irfan view v. 4.41 and Image J v. 1.51 software.

**Antibiotic treatment**. We attempted to eliminate endosymbiotic bacteria by incubation for 2 weeks in Hemi medium supplemented with chloramphenicol (100 μg∙ml^-1^), ampicillin (100 μg∙ml^-1^), kanamycin (50 μg∙ml^-1^), gentamycin (5 μg∙ml^-1^), azithromycin (50 μg∙ml^-1^) or 20 μl∙ml^-1^ penicillin-streptomycin-neomycin cocktail (Sigma-Aldrich). Sub-cultures were incubated in antibiotic-free medium for 2 days and subsequently analyzed by FISH with EUB338 probe. Cultures starved for 2 weeks in seawater were also analyzed.

**DNA isolation and amplification**. Total genomic DNA of diplonemids and endosymbionts from axenic cultures was isolated using DNeasy Blood & Tissue Kit (Qiagen) following the manufacturer’s protocol A. Almost full-length 18S rRNA gene was PCR amplified with universal eukaryotic primers SA (AACCTGGTTGATCCTGCCAGT) and SB (TGATCCTCCTGCAGGTCCACCT). Endosymbiont 16S rRNA gene was amplified with bacteria-specific forward (GCTTAACACATGCAAG) and reverse primers (CATTGTAGCACGTGT), yielding approximately 1180 bp-long amplicons, which were sequenced.

**Cultivation of endosymbionts and infection experiments**. Trophic cells form 10 ml cultures were pelleted and re-suspended in 2 ml of fresh Hemi medium. The host cells were broken by repetitive passage through a 23G syringe needle. For cultivation of endosymbionts, the lysed host cells were subsequently inoculated into fresh Hemi medium, filter-sterilized marine broth (Difco), or plated onto marine broth agar or Hemi medium agar.

In order to investigate possible horizontal transmission of endosymbiotic bacteria into taxonomically related hosts, cultures of *D. japonicum* or *D. aggregatum* were co-cultured (1:1) for 10 days with endosymbiont-free diplonemids, namely *Lacrimella lanifica*, *Sulcionema specki*, and Hemistasiidae YPF1610*.* Apart from it, these endosymbiont-free diplonemids, as well as three other diplonemid species (*Rhynchopus serpens*, *Rhynchopus humris*, and *Flectonema neradi*) were mixed with bacterial pellets of *D. japonicum* and *D. aggregatum* obtained after mechanical breakage of cells, and were regularly cultured for 7 days. Possible infection was verified by FISH.

**Phylogenetic analyses**. To put the newly described diplonemids in a proper evolutionary context, we have collected all available 18S rRNA sequences of diplonemids using EukRef approach (eukref.org). Since the preliminary analysis showed close affiliation of *D. japonicum* and *D. aggregatum* with the genus *Diplonema*, we have focused our sampling on members of family Diplonemidae sensu Yabuki and Tame, 2015 (56) and Tashyreva et al., 2018 (7). Sequences of eupelagonemids and *Hemistasia*-related diplonemids were used as an outgroup. The resulting dataset comprised of 74 sequences. 16S rRNA sequences of bacterial endosymbionts showed alpha-proteobacterial affiliation when blasted against nr database of NCBI. They were therefore added to the dataset with comprehensive sampling of alpha-proteobacteria and several beta- and gamma-proteobacteria as outgroup (179 sequences).

Sequences in both dataset sequences were aligned using the LOCALPAIR algorithm implemented in MAFFT (57) and the gap-rich and ambiguously aligned regions were removed by eye in Seaview 4 (58). Maximum likelihood analysis under the gamma-corrected GTR model was then carried out by RAxML 8.2.4 (59). The best-scoring topology was inferred from 100 randomized starting trees. Non-parametric bootstrap values as a mean of branching support were then inferred from 1000 replicates using thorough algorithm under the model specified above in RAxML. Robustness of observed topologies was also tested using Bayesian posterior probabilities as inferred by Phylobayes 4.1 (60) under the empirical mixture model C40 combined with exchange rates as defined by GTR matrix (C40+GTR model). Two independent MCMC chains were ran until convergence was reached (i.e. maximum observed discrepancy was lower than 0.1 and effective sample size of observed statistics was at least 100).

**Electron microscopy**. For scanning electron microscopy (SEM), *D*. *japonicum* pellets were placed in seawater-based Parducz’s fixative (pH 7.4), instantly frozen in liquid nitrogen and freeze-dried at -60°C under high vacuum for 12 hours (61). *D. aggregatum* cells were fixed in 2.5% glutaraldehyde in either 0.1 M phosphate (pH 7.2) or 0.3 M sodium cacodylate buffer (pH 7.3), post-fixed in 1% OsO_4_, dehydrated through a graded acetone or ethanol series (30%-100%), and then dried at the critical point using CO_2_ or hexamethyldisilazane, respectively. The samples were mounted on a SEM specimen stub, coated with gold in Sputter Coater Polaron chamber, and observed with a JEOL JSM-7401-F microscope at an accelerating voltage of 4 kV. Samples for transmission electron microscopy (TEM) were processed by high pressure freezing technique according to the procedure described elsewhere (62). Observations were performed on JEOL 1010 transmission electron microscope at accelerating voltage of 80 kV.

References

54. Zíková A, Schnaufer A, Dalley RA, Panigrahi AK, Stuart KD. 2009. The F0F1-ATP synthase complex contains novel subunits and is essential for procyclic *Trypanosoma brucei*. PLoS Pathog 5:e1000436.

55. Týč J, Klingbeil MM, Lukeš J. 2015. Mitochondrial heat shock protein machinery Hsp70/Hsp40 is indispensable for proper mitochondrial DNA maintenance and replication. mBio 6:e02425-14.

56. Yabuki A, Tame A. 2015. Phylogeny and reclassification of *Hemistasia phaeocysticola* (Scherffel) Elbrächter & Schnepf, 1996. J Eukaryot Microbiol 62:426–429.

57. Katoh K, Standley DM. 2013. MAFFT Multiple sequence alignment software version 7: improvements in performance and usability. Mol Biol Evol 30:772–780.

58. Gouy M, Guindon S, Gascuel O. 2010. SeaView Version 4: a multiplatform graphical user interface for sequence alignment and phylogenetic tree building. Mol Biol Evol 27:221–224.

59. Stamatakis A. 2014. RAxML version 8: a tool for phylogenetic analysis and post-analysis of large phylogenies. Bioinformatics 30:1312–1313.

60. Lartillot N, Lepage T, Blanquart S. 2009. PhyloBayes 3: a Bayesian software package for phylogenetic reconstruction and molecular dating. Bioinformatics 25:2286–2288.

61. Small EB, Marszalek DS. 1969. Scanning electron microscopy of fixed, frozen, and dried protozoa. Science (80) 163:1064–1065.

62. Yurchenko V, Votýpka J, Tesařová M, Klepetková H, Kraeva N, Jirků M, Lukeš J. 2014. Ultrastructure and molecular phylogeny of four new species of monoxenous trypanosomatids from flies (Diptera: *Brachycera*) with redefinition of the genus *Wallaceina*. Folia Parasitol 61:97–112.
