# Supplementary material for: Life Cycle, Ultrastructure, and Phylogeny of New Diplonemids and Their Endosymbiotic Bacteria
Source: mBio. 2018 Mar 6;9(2):e02447-17. doi: 10.1128/mBio.02447-17 (PMC5845003; doi:10.1128/mBio.02447-17)
Supplement: TABLE S1 [file mbo001183766st1.docx]

Table S1.

| Trait | *D. japonicum* | *D. aggregatum* | *D. ambulator* | *D. papillatum* | *D. nigricans* | *D. metabolicum* | *D. breviciliata* |
| --- | --- | --- | --- | --- | --- | --- | --- |
| Cell size, μm | 15.3-23 × 4.5-7 | 17.6-24.7 × 4.3-6 | 17-24 × 6-10 | 10-24 × 4-8 | 40-50 × 3-7 | 30-48 × ND | 28-35 × 8-10 |
| Shape | cylindrical or tapering posteriorly | narrow anterior, round posterior ends | cylindrical tapering anteriorly | variable | broad anterior end | conical tapering posteriorly | narrow anterior, round posterior ends |
| Flagellar pocket | subapical | subapical | subapical | apical | subapical | apical | subapical |
| Swimming stage | yes | yes | ND | ND | ND | ND | ND |
| Paraxonemal rods | only in swimming and sessile stages | only in swimming and sessile stages | absent | absent | absent | ND | ND |
| Flagella* | equal to subequal | unequal | equal to subequal | equal to subequal | ND | ND | equal |
| Extrusomes | present | present | ND | ND | present | ND | ND |
| Pigment production | none | none | present | none | present | ND | ND |
| Movement | gliding, flexing flagella, metabolic, fast swimming | gliding, flexing flagella, metabolic, fast swimming | gliding, flexing flagella, metabolic | swimming, metabolic | gliding, flickering flagella motion, metabolic | writhing motion, metabolic | metabolic |

* – in trophic stage

ND – not described
